# From Conventional to Intelligent Triage: A Systematic Review of Artificial Intelligence and Machine Learning Applications in Emergency Departments

**DOI:** 10.7759/cureus.111866

**Published:** 2026-07-01

**Authors:** Karar Omer Karar Bala, Rasha Abdelrahman Musharaf Abdelrahman, Eslam Abdelkawy, Ali Albalushi, Mohamed Abdalla Mohamed ElshikhIdris, Omer Kamal Omer, Hanady ME M Osman

**Affiliations:** 1 Emergency Medicine, University of Medical Sciences and Technology, Khartoum, SDN; 2 Urgent Care, Mediclinic Al Ain Hospital, Al Ain, ARE; 3 General Internal Medicine, George Eliot Hospital, Nuneaton, GBR; 4 Pediatric Emergency Medicine, Sohar Hospital, Sohar, OMN; 5 General Practice, Al Yarmouk College, Khartoum, SDN; 6 Orthopedic Surgery, Luton Hospital, Luton, GBR; 7 Quality Improvement and Patient Safety, Najran Armed Forces Hospital, Ministry of Defense Health Services, Najran, SAU

**Keywords:** artificial intelligence, emergency department, machine learning, systematic review, triage, xgboost

## Abstract

Conventional triage systems in emergency departments (EDs) face limitations including subjectivity and variability. Artificial intelligence (AI) and machine learning (ML) offer potential solutions. This systematic review evaluates AI/ML models for ED triage, focusing on model types, performance, and comparisons with conventional systems.

A systematic search of PubMed, Scopus, Web of Science, and Embase was conducted (January 2021-April 2025). Studies applying AI/ML for ED triage with reported performance metrics were included. The Preferred Reporting Items for Systematic Reviews and Meta-Analyses (PRISMA) guidelines were followed, and the Prediction Model Risk of Bias Assessment Tool (PROBAST) was used for risk of bias assessment. The protocol was prospectively registered in the International Prospective Register of Systematic Reviews (PROSPERO) (registration number: CRD420261407520). Narrative synthesis was performed.

Eight retrospective or cross-sectional studies were included (n=2,000 to >2.6 million patients). XGBoost was the most consistently top-performing model (AUC: 0.76-0.96). Natural language processing (NLP) improved predictive accuracy when added to structured data. AI/ML outperformed conventional triage scales in direct comparisons (e.g., area under the receiver operating characteristic curve (AUROC) 0.991 vs. 0.844 for pediatric critical illness). PROBAST showed low bias in six studies, unclear in one, and high in one. No prospective implementation studies were identified.

AI/ML models, especially XGBoost and NLP-enhanced architectures, show strong predictive performance for ED triage outcomes. However, retrospective designs, lack of external validation, and absent prospective data limit current evidence. Rigorous implementation research is needed before clinical adoption.

## Introduction and background

Emergency departments (EDs) are critical components of healthcare systems, providing immediate care for patients with a wide range of acute conditions [1]. Efficient triage, defined as the process of prioritizing patients according to the severity and urgency of their condition, is essential in these settings to ensure timely intervention and optimal use of limited healthcare resources. However, EDs globally continue to face challenges such as overcrowding, increasing patient complexity, and variability in clinical decision-making, all of which can compromise the effectiveness of traditional triage systems [2].

Conventional triage methods, including the Emergency Severity Index (ESI) [3], Manchester Triage System (MTS) [4], and Canadian Triage and Acuity Scale (CTAS) [5], primarily rely on structured algorithms combined with clinician judgment [6]. For example, in the ESI system, patients are categorized into five levels based on the urgency of their condition and anticipated resource needs. While these tools have standardized patient prioritization to some extent, they remain limited by inter-observer variability, subjectivity, and reliance on a restricted set of clinical parameters [7]. In fast-paced emergency settings, such limitations may contribute to under-triage or over-triage, potentially affecting patient outcomes and resource utilization.

In recent years, artificial intelligence (AI), which refers to computer systems designed to perform tasks that typically require human intelligence, and machine learning (ML), a subset of AI that enables systems to learn patterns from data and make predictions, have emerged as promising tools to enhance triage processes in emergency care [8]. These approaches can analyze large and complex datasets, including electronic health records, vital signs, laboratory results, and imaging data, to identify patterns associated with clinical deterioration or adverse outcomes [9]. Various ML models, such as logistic regression, random forests, gradient boosting machines, and deep learning networks, have demonstrated improved predictive performance in identifying patient acuity, risk of admission, and mortality compared to traditional scoring systems [10].

Despite their potential, the clinical integration of AI/ML-based triage systems remains limited due to challenges related to data quality, model interpretability, external validation, and workflow integration. Moreover, heterogeneity in study designs and performance metrics makes it difficult to draw definitive conclusions regarding their effectiveness. Therefore, this systematic review aims to critically evaluate the application of AI and ML in ED triage, focusing on model types, data inputs, predictive performance, and reported clinical outcomes, while identifying current gaps and future research directions.

## Review

Methodology

Study Design

This systematic review was conducted in accordance with the Preferred Reporting Items for Systematic Reviews and Meta-Analyses (PRISMA) guidelines [11] to ensure transparency, reproducibility, and methodological rigor. The review protocol was prospectively registered in the International Prospective Register of Systematic Reviews (PROSPERO) (registration number: CRD420261407520) prior to data extraction. The review protocol was structured to comprehensively identify, evaluate, and synthesize evidence on the application of AI and ML in ED triage systems.

Eligibility Criteria (PICOS Framework)

Studies were selected based on the PICOS framework [12]. The population (P) included patients presenting to EDs across all age groups. The intervention (I) consisted of AI- or ML-based models applied for triage or risk stratification. Comparators (C) included conventional triage systems, clinician judgment, or existing scoring tools such as the ESI [3], MTS [4], or other standard approaches. Outcomes (O) included triage accuracy, predictive performance metrics (such as area under the curve (AUC), sensitivity, specificity, and accuracy), as well as clinically relevant outcomes including hospital admission, intensive care unit (ICU) transfer, or mortality prediction. Study designs (S) included original observational studies, retrospective or prospective cohort studies, and model development or validation studies. Reviews, editorials, conference abstracts without full text, and studies not focused on ED triage or not involving AI/ML-based approaches were excluded. To ensure the inclusion of the most up-to-date evidence, only studies published between 2021 and 2025 were considered. This time frame was selected to ensure the inclusion of the most recent and clinically relevant evidence reflecting the rapid evolution of AI/ML methodologies in emergency medicine.

Information Sources and Search Strategy

A comprehensive literature search was performed in four major electronic databases: PubMed, Scopus, Web of Science, and Embase. These databases were selected due to their extensive coverage of biomedical, clinical, and computational research. The search strategy combined keywords and Medical Subject Headings (MeSH) related to "emergency department", "triage", "artificial intelligence", "machine learning", and "deep learning", along with relevant Boolean operators. The search was limited to studies published in English between January 2021 and April 2025. The full search strategy for each database is provided in the Appendices. 

Study Selection

All identified records were imported into EndNote X21 (Clarivate, London, United Kingdom) for reference management. Duplicate records were systematically removed using the software's automated and manual deduplication functions. Following this, two independent reviewers screened titles and abstracts for relevance based on the predefined eligibility criteria. Full-text articles of potentially eligible studies were then assessed in detail. Any disagreements between reviewers were resolved through discussion and consensus and, when necessary, consultation with a third reviewer.

Data Extraction

Data from the included studies were extracted using a standardized extraction framework. Extracted information included study characteristics (author, year, country, and design), patient population, data sources, type of AI/ML model used, comparator methods, outcomes assessed, and key performance metrics. Additional details such as sample size and type of triage setting were also recorded to allow for comprehensive comparison across studies.

Risk of Bias Assessment

The risk of bias in the included studies was assessed using the Prediction Model Risk of Bias Assessment Tool (PROBAST) [13]. This tool is specifically designed to evaluate the quality and applicability of studies developing or validating prediction models, and it assesses risk of bias across four key domains: participants, predictors, outcomes, and analysis. Each included study was independently evaluated by two reviewers to ensure consistency and reduce subjective judgment, and any disagreements were resolved through discussion and consensus. PROBAST was chosen for this review because of its established suitability for critically appraising diagnostic and prognostic prediction models, particularly those commonly applied in AI- and ML-based triage and clinical decision support research.

Data Synthesis and Justification for Narrative Approach

A narrative synthesis was adopted, and meta-analysis was deliberately not undertaken due to substantial clinical, methodological, and statistical heterogeneity across the eligible studies, which rendered quantitative pooling statistically inappropriate.

Clinically, the studies spanned adult, pediatric, and mixed ED populations across seven healthcare systems, addressing distinct questions including triage acuity, hospital admission, ICU/critical illness prediction, electrocardiogram (ECG) necessity, and respiratory infection risk. Methodologically, they employed different model architectures (logistic regression (LR), tree-based ensembles, deep learning/natural language processing (NLP) networks), predictor sets, and validation strategies. Statistically, outcome definitions and metrics were incommensurable (AUC/area under the receiver operating characteristic curve (AUROC), area under the precision-recall curve (AUPRC), accuracy, sensitivity, specificity, F1), with no single common effect estimate available for pooling.

Sample sizes also varied by more than three orders of magnitude (approximately 2,000 to over 2.6 million patients), reflecting fundamentally different data regimes. Pooling across such disparate scales and designs would be numerically dominated by the largest registry studies and would generate a spuriously precise summary statistic. For these reasons, results were synthesized descriptively, structured around model type, predictor inputs, outcomes, comparator use, and methodological quality. No pooled effect estimates, p-values, or 95% confidence intervals (CI) were generated.

Results

Study Selection Process

**Figure 1:**
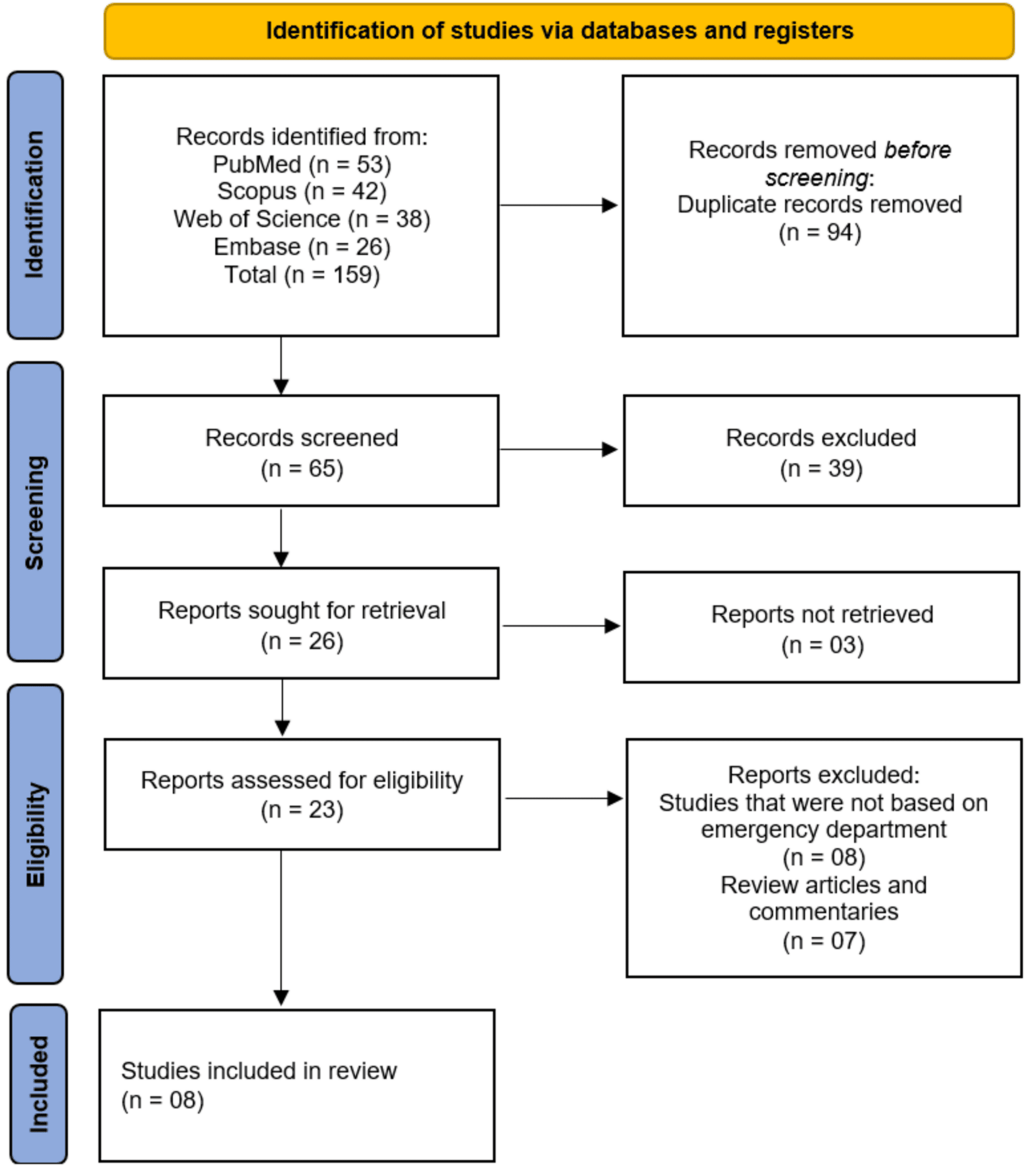
PRISMA flowchart illustrating the study selection process PRISMA: Preferred Reporting Items for Systematic Reviews and Meta-Analyses

The study selection process followed the PRISMA guidelines, as summarized in the PRISMA flow diagram. A total of 159 records were identified through four electronic databases: PubMed (n=53), Scopus (n=42), Web of Science (n=38), and Embase (n=26). Prior to screening, 94 duplicate records were removed, leaving 65 records for title and abstract screening. Of these, 39 records were excluded based on irrelevance to the review question or inappropriate study design. The remaining 26 reports were sought for full-text retrieval, of which three could not be obtained. Consequently, 23 reports were assessed for full-text eligibility. Following detailed evaluation, 15 reports were excluded for the following reasons: eight studies were not based on ED settings, and seven were review articles or commentaries that did not present original research. Ultimately, eight studies [14-21] met the inclusion criteria and were included in this systematic review (Figure 1).

Key Characteristics

A total of eight studies meeting the inclusion criteria were synthesized in this systematic review. The characteristics of these studies, including country, study design, ED setting, sample size, data sources, AI/ML models, and primary objectives, are summarized in Table 1.

**Table 1:** Characteristics of the included studies AUH: Asia University Hospital; CMUH: China Medical University Hospital; CRP: C-reactive protein; CTNs: chief triage notes; CXR: chest X-ray; DL: deep learning; ED: emergency department; EHR: electronic health record; ICD-10: International Classification of Diseases, 10th Revision; ML: machine learning; NEDIS: National Emergency Department Information System; pts: patients

Author (year)	Country	Study design	ED setting	Sample size	Data source
Ellertsson et al. (2023) [14]	Iceland	Retrospective	Adult (primary-care triage)	2,000 CTNs (1,915 pts)	EHR (clinical text, CRP, CXR)
Kırelli (2026) [15]	Turkey	Retrospective ML/DL study	Mixed	7,000	EHR (vitals + text)
Yıldırım et al. (2025) [16]	Turkey	Retrospective ML study	Mixed	359,000 visits (128k pts)	EHR (vitals, demographics, text)
Chang et al. (2022) [17]	Taiwan	Retrospective multicenter study	Adult	44,775 (CMUH) + 16,047 (AUH)	EHR (demographics, vitals, triage, clinical variables)
Hwang and Lee (2022) [18]	South Korea	Cross-sectional registry study	Pediatric	2,621,710	NEDIS (EHR/vitals)
Klang et al. (2021) [19]	USA	Retrospective cohort (development + validation)	Adult	412,858	EHR (structured + clinical notes)
Lee et al. (2021) [20]	Taiwan	Retrospective cohort	Mixed ED	282,971 visits	EHR, vitals, ICD-10, triage system
Tsai et al. (2022) [21]	Taiwan	Retrospective cohort	Adult	301,658 visits	EHR (triage, vitals, complaints)

The corresponding performance metrics, input variables, predicted outcomes, comparisons with standard triage, and key findings are presented in Table 2. The studies spanned multiple countries: two from Turkey [15,16], three from Taiwan [17,20,21], and one each from Iceland [14], South Korea [18], the USA [19], and Taiwan [20]. All but one study employed a retrospective design [14-17,19-21]; one used a cross-sectional registry design [18]. Sample sizes varied considerably, ranging from approximately 2,000 clinical encounters [14] to over 2.6 million pediatric visits [18], reflecting the large-scale data typically required for AI/ML model development in emergency triage.

**Table 2:** AI/ML performance and clinical outcomes in triage systems AI: artificial intelligence; Acc: accuracy; AUC: area under the curve; AUPRC: area under the precision-recall curve; AUROC: area under the receiver operating characteristic curve; BERT: Bidirectional Encoder Representations from Transformers; BMI: body mass index; BoW: bag-of-words; BP: blood pressure; CTAS: Canadian Triage and Acuity Scale; CXR: chest X-ray; DNN: deep neural network; DT: decision tree; ECG: electrocardiogram; ESI: Emergency Severity Index; F1: F1 score; HR: heart rate; LASSO: Least Absolute Shrinkage and Selection Operator; LR: logistic regression; LRTI: lower respiratory tract infection; LSTM: long short-term memory; MAP: mean arterial pressure; ML: machine learning; MLP: multilayer perceptron; NLP: natural language processing; NPV: negative predictive value; NSICU: neurological/neurosurgical intensive care unit; pedKTAS: Pediatric Korean Triage and Acuity Scale; PPV: positive predictive value; Rec: recall; RF: random forest; RR: respiratory rate; RSTM: Regularized Sparse Topic Model; Sens: sensitivity; SHAP: SHapley Additive exPlanations; Spec: specificity; TTAS: Taiwan Triage and Acuity Scale; W-F1: weighted F1 score; XGBoost: extreme gradient boosting

Author (year)	AI/ML model	Input variables	Outcome predicted	Performance metrics	Comparison with standard triage
Ellertsson et al. (2023) [14]	RSTM (LASSO + DNN features)	Patient symptoms (pre-consult), ~50 features (SHAP)	LRTI risk score; low vs. high risk groups	Sens 0.74, Spec 0.40 (antibiotics); Sens up to 1.0 (CXR); no AUC	No direct ESI/CTAS comparison; aligned with clinical outcomes
Kırelli (2026) [15]	XGBoost (best; also RF, LSTM, CNN)	Age, vitals (BP, pulse, temp), gender, complaint text (Word2Vec NLP)	3-level triage (red/yellow/green)	AUC up to 0.963; Acc 0.85; Sens 0.85; F1 0.82	No formal ESI comparison; improved consistency over clinician-based triage
Yıldırım et al. (2025) [16]	XGBoost (best model)	Age, vitals, gender, arrival type, chief complaint + engineered + embeddings (Word2Vec/BERT)	3-level triage (green/yellow/red)	Acc 81.52%, AUC 86.66%, Rec 63.29%, F1 66.11%, W-F1 68.69%	No direct ESI/CTAS comparison
Chang et al. (2022) [17]	CatBoost, XGBoost, RF, DT, LR	32 ED triage features (vitals, age, BMI, chief complaint, arrival/mode, comorbidities, transfer)	Short ED stay (<4 h)	Best: XGBoost AUC 0.761; CatBoost AUC 0.755; LR lowest AUC 0.694	No direct ESI/CTAS comparison
Hwang and Lee (2022) [18]	RF	Age, vitals, consciousness, demographics, time variables	Critical illness and hospitalization	AUROC 0.991 (critical), 0.943 (admission); AUPRC up to 0.729	Outperformed pedKTAS (AUROC 0.991 vs. 0.844; 0.943 vs. 0.680)
Klang et al. (2021) [19]	XGBoost (EHR + BoW text model)	Demographics, vitals, history, ED visits, triage notes	NSICU admission (30 min)	AUC 0.93 (0.92-0.95); tabular 0.92; text 0.90; Sens 58% @ 99% spec	Outperformed ESI-related variables (AUC up to 0.85)
Lee et al. (2021) [20]	Neural network (3-layer MLP, batch norm, sigmoid)	Age, sex, vitals (HR, RR, BP, MAP, temp), medical history, chief complaint score	Admission (yes/no)	AUC 0.80; Sens 0.67; Spec 0.78; PPV 0.37; NPV 0.93	Compared with TTAS
Tsai et al. (2022) [21]	LR, DT, RF, XGBoost (best: XGBoost)	Age, sex, vitals, BMI, triage level, 404 chief complaints	ECG within 2 hours	AUC 0.845-0.891; Sens ~0.81; Spec ~0.81; PPV 0.66-0.71; NPV 0.89	Triage level used as a predictor

AI/ML Models and Input Variables

Across the eight studies, a wide range of ML and deep learning architectures were applied. Traditional models such as LR, decision trees (DT), random forest (RF), support vector machines (SVM), and k-nearest neighbors (KNN) were commonly compared alongside gradient-boosted methods like XGBoost and CatBoost [15-17,21]. Deep learning approaches included deep neural networks (DNNs) [14,20], convolutional neural networks (CNNs), long short-term memory (LSTM) networks [15,16], and combined CNN-LSTM-attention mechanisms [16]. NLP techniques, such as Word2Vec, BioBERT, and bag-of-words, were increasingly integrated to process unstructured chief complaints or triage notes [15,16,19]. Input variables typically included demographics, vital signs (e.g., blood pressure, pulse, temperature, respiratory rate), triage level, chief complaint, and medical history, with data sources drawn from electronic health records (EHRs), imaging, and structured triage systems [14-21].

Performance of AI/ML Models for Triage Prediction

In predicting multi-level triage urgency (e.g., green/yellow/red), Kırelli [15] reported that XGBoost achieved the highest AUC of 0.963, with an accuracy of 0.85 and a sensitivity of 0.85, outperforming other models including RF, LSTM, and CNN. Similarly, Yıldırım et al. [16] found XGBoost to be the best performer among LR, RF, NN, and sequential deep learning models, achieving an accuracy of 81.52%, an AUC of 86.66%, and a weighted F1 score of 68.69%; they noted that sequential data and red-class predictions remained challenging. For predicting critical illness and hospitalization in pediatric ED patients, Hwang and Lee [18] reported outstanding performance using RF (AUROC 0.991 for critical illness and 0.943 for admission), significantly outperforming the conventional Pediatric Korean Triage and Acuity Scale (pedKTAS) [22] (AUROC 0.844 and 0.680, respectively).

Prediction of Admission, ICU Need, and Clinical Outcomes

Several studies focused on disposition-related outcomes. Lee et al. [20] used a three-layer neural network to predict hospital admission from triage data, achieving an AUC of 0.80, a sensitivity of 0.67, a specificity of 0.78, and a high negative predictive value of 0.93. Chief complaint was identified as the most important predictor, and performance remained stable with reduced data volumes, though it was weaker in pediatric subgroups. Klang et al. [19] developed an XGBoost model combining structured EHR data with bag-of-words from triage notes to predict neuroscience intensive care unit (NSICU) admission within 30 minutes of ED arrival, achieving an AUC of 0.93 (95% CI 0.92-0.95). This combined model outperformed tabular-only (AUC 0.92) or text-only (AUC 0.90) models and significantly exceeded triage-level-based predictions (AUC up to 0.85). For low-severity patients, Chang et al. [17] applied multiple ML models to predict ED length of stay <4 hours, with XGBoost (AUC 0.761) and CatBoost (AUC 0.755) performing best; vital signs and chief complaint were the most influential features.

ECG Recommendation and Respiratory Infection Risk

Tsai et al. [21] developed an AI-based ECG recommendation system predicting the need for an ECG within two hours of ED arrival. XGBoost outperformed LR, DT, and RF, yielding an AUC of 0.845-0.891, a sensitivity and a specificity of around 0.81, and robust performance across COVID-19 and non-COVID-19 cohorts. Triage level, age, and chest pain were the strongest predictors. In a primary-care triage setting for respiratory symptoms, Ellertsson et al. [14] used RSTM (LASSO LR) with DNN features to predict lower respiratory tract infection (LRTI) risk. Sensitivity for antibiotic prescription was 0.74 with a specificity of 0.40, and for chest radiography, sensitivity reached 1.0. High-risk patients had worse clinical outcomes, while low-risk patients had no pneumonia, suggesting the potential to reduce unnecessary testing.

Comparison With Conventional Triage Systems

When comparisons with standard triage scales, such as the ESI [3], CTAS [5], Taiwan Triage and Acuity Scale (TTAS) [23], and pedKTAS [22], were performed, AI/ML models consistently demonstrated superior or complementary discrimination. Hwang and Lee [18] showed that RF markedly outperformed pedKTAS for both critical illness (AUROC 0.991 vs. 0.844) and hospitalization (0.943 vs. 0.680). Klang et al. [19] reported that their combined XGBoost model outperformed ESI-related variables (AUC up to 0.93 vs. 0.85). Kırelli [15] noted that XGBoost improved consistency over clinician-based triage. Although several studies did not directly compare AI/ML to formal triage scales [14,16,17], their models achieved clinically useful accuracy for risk stratification and resource allocation. Overall, the evidence indicates that AI/ML models, particularly gradient-boosted ensembles and deep learning with NLP, can enhance the prediction of triage levels, critical illness, admission, ICU need, and ED efficiency metrics compared to conventional approaches. However, variability in outcome definitions, settings, and direct comparator use warrants cautious interpretation.

Risk of Bias Assessment

Six studies demonstrated a low overall risk of bias [14-16,19-21], each scoring low across all four individual domains, indicating robust methodological quality in participant selection, predictor definition, outcome ascertainment, and statistical analysis. Chang et al. [17] received an unclear overall rating due to insufficient reporting in the analysis domain, particularly regarding handling of missing data and model selection procedures, though applicability remained low. Hwang and Lee [18] were assessed as having a high overall risk of bias, driven by a high-risk rating in the analysis domain; despite low risk in participants, predictors, and outcome domains, issues such as lack of calibration reporting and incomplete description of missing data handling contributed to this judgment. Applicability concerns were low across all eight studies [14-21], meaning that the included populations, predictors, and outcomes were appropriately matched to the review question and clinical ED triage setting. Overall, the majority of studies were methodologically sound, though future research should address transparent reporting of analytical procedures, particularly calibration and missing data handling, to further reduce bias (Table 3).

**Table 3:** Risk of bias assessment of the included studies using PROBAST PROBAST: Prediction Model Risk of Bias Assessment Tool

Author (year)	Domain 1: participants	Domain 2: predictors	Domain 3: outcome	Domain 4: analysis	Overall risk of bias	Applicability concern
Ellertsson et al. (2023) [14]	Low	Low	Low	Low	Low	Low
Kırelli (2026) [15]	Low	Low	Low	Low	Low	Low
Yıldırım et al. (2025) [16]	Low	Low	Low	Low	Low	Low
Chang et al. (2022) [17]	Low	Low	Low	Unclear	Unclear	Low
Hwang and Lee (2022) [18]	Low	Low	Low	High	High	Low
Klang et al. (2021) [19]	Low	Low	Low	Low	Low	Low
Lee et al. (2021) [20]	Low	Low	Low	Low	Low	Low
Tsai et al. (2022) [21]	Low	Low	Low	Low	Low	Low

Discussion

This systematic review synthesized evidence from eight studies evaluating the application of AI and ML models for triage in ED settings. The findings consistently demonstrate that AI/ML models, particularly gradient-boosted ensembles such as XGBoost and deep learning architectures incorporating NLP, can achieve high discriminatory performance for predicting triage urgency, hospital admission, ICU need, critical illness, and ED efficiency metrics. Across studies, XGBoost emerged as the most frequently top-performing model [15-17,21], often outperforming traditional LR and even some deep learning approaches. Our review also found that integrating unstructured text data from chief complaints and triage notes consistently improved predictive accuracy compared to using structured data alone [15,16,19], highlighting the value of NLP in capturing nuanced clinical presentations that may not be fully encoded in structured fields. However, despite promising performance metrics, the field is characterized by substantial heterogeneity in outcome definitions, triage scales, and comparator methods, and only a minority of studies directly compared AI/ML predictions with conventional triage systems such as the ESI or the pedKTAS [18,19].

Predictive Model Performance and Algorithmic Trends

The finding that XGBoost frequently outperformed other models in ED triage prediction is consistent with a growing body of evidence outside our review. For instance, Raita et al. [24] applied four ML approaches, that is, Lasso regression, RF, a gradient-boosted DT, and DNN, to routinely available triage data from 135,470 adult ED visits and found that all of them outperformed a conventional ESI-based reference model for predicting both critical care (AUC up to 0.86 vs. 0.74) and hospitalization (AUC up to 0.82 vs. 0.69) outcomes, with the gradient-boosted and deep learning models among the strongest performers. Likewise, Levin et al. [25] developed an RF-based electronic triage system using routinely collected triage variables across multiple urban and community EDs (172,726 visits) and showed that it differentiated patients by clinical outcome more accurately than the ESI. These performance levels are broadly consistent with the range observed in our included studies (AUC 0.80-0.96). The consistency with which gradient-boosted DT perform well across independent datasets and clinical contexts suggests that they are particularly well-suited to the tabular, heterogeneous, and often incomplete data typical of ED triage encounters, owing to their robustness to outliers, ability to model nonlinear interactions, and built-in handling of missing values.

Role of NLP in Triage Enhancement

Our review also highlighted the added value of NLP for extracting predictive signals from unstructured chief complaints and triage notes. Kırelli [15] demonstrated that incorporating Word2Vec-encoded complaint text improved critical case detection, while Klang et al. [19] found that a combined structured-plus-text model outperformed either modality alone. These findings are reinforced by external studies. For example, Sterling et al. [26] applied NLP to nursing triage notes from more than 250,000 ED encounters and showed that text-derived representations of these free-text notes, used in isolation from structured data, predicted final ED disposition (admission, transfer, or in-ED death) with an AUC of up to approximately 0.79, demonstrating that triage narratives alone carry substantial predictive signal. Collectively, these external findings support our conclusion that NLP is not merely an incremental improvement but a substantial addition to ED triage prediction, enabling models to leverage the rich, unstructured narrative data that clinicians already routinely collect.

Clinical Outcomes and Risk Stratification Performance

Regarding the prediction of critical illness and disposition, Hwang and Lee [18] achieved exceptional performance (AUROC 0.991 for critical illness) using RF in a pediatric population, significantly outperforming conventional pedKTAS. This magnitude of improvement is notable but warrants context. An external study by Park et al. [27] evaluated the Korean Triage and Acuity Scale (KTAS) in a general adult ED population and found that augmenting it with the National Early Warning Score improved the prediction of serious adverse events, including ICU admission and mortality, raising the AUC from 0.75 (KTAS alone) to 0.81 for the combined model, a real but considerably more modest gain than the improvement reported by Hwang and Lee [18]. The difference may be explained in part by the very large sample size in Hwang and Lee [18] (over 2.6 million visits), which likely provided stable estimates of rare critical events, and by the inclusion of more granular clinical features beyond the limited KTAS variables. However, the high risk of bias we identified in that study, specifically the lack of calibration reporting and unclear missing data handling, suggests that the reported AUROC may be optimistic. Calibration, which assesses whether predicted probabilities match observed event rates, is critically important for clinical decision support; an overconfident model could lead to under-triage of high-risk patients.

Operational Metrics and Workflow Optimization in ED Settings

Our review also identified studies predicting ED operational outcomes, such as short length of stay [17] and ECG necessity within two hours [21]. These applications represent an important extension beyond clinical risk prediction toward resource allocation and workflow optimization. Chang et al. [17] found that vitals and chief complaint were the most important predictors for identifying low-severity patients likely to discharge within four hours, which could facilitate fast-track protocols. Similarly, Tsai et al. [21] developed an XGBoost model to determine which patients need an ECG within two hours, potentially reducing unnecessary testing and streamlining cardiac evaluation. These findings align with the broader literature on AI for ED operations. For example, Hong et al. [28] used ML, including a gradient-boosted (XGBoost) model, on more than half a million ED visits to predict hospital admission at the point of triage, achieving an AUC of approximately 0.87 and showing that adding patient historical data to information collected at triage significantly improved predictive performance. Accurate early prediction of admission of this kind can, in turn, support downstream bed management and resource planning.

Collectively, these findings suggest that AI/ML systems in emergency care operate across three interconnected domains: operational metrics (workflow efficiency and resource utilization), predictive clinical models (risk stratification and diagnosis), and validation frameworks (internal and external performance assessment). However, while predictive performance is frequently reported, operational utility and real-world implementation remain underexplored. Importantly, operational models require continuous recalibration, integration with live ED information systems, and adaptation to temporal shifts in patient flow, requirements that were not addressed in the included studies.

Barriers to Clinical Adoption and Implementation Gap

Despite strong retrospective performance, several barriers limit clinical translation. First, external validation remains scarce, with most studies relying on single-center retrospective datasets. This raises concerns about generalizability across diverse ED populations, workflows, and healthcare systems. Second, ongoing model calibration and performance monitoring are rarely reported, despite known risks of model drift in dynamic ED environments. Third, integration into real-time clinical workflows remains a major challenge, as AI outputs must align with triage nurse decision-making and avoid disrupting established protocols. Fourth, equity considerations are largely absent; few studies evaluated whether models perform consistently across age, sex, or comorbidity subgroups, raising concerns about algorithmic bias in high-stakes triage decisions. These challenges highlight a critical gap between technical performance and clinical deployment. Without robust external validation, recalibration strategies, and implementation studies assessing clinician acceptance and workflow integration, the clinical utility of these models remains limited.

Comparative Performance With Conventional Triage Systems

Despite the generally strong performance of AI/ML models in our review, only three studies [18,19,21] directly compared AI/ML predictions to conventional triage scales (pedKTAS, ESI-related variables, or triage level) rather than simply reporting model performance in isolation. Direct head-to-head comparisons are essential to determine whether AI/ML offers added value beyond existing, well-established triage systems. A systematic review by Askar et al. [29], which synthesized 116 ML studies predicting all-cause hospital admission and readmission in adults, highlighted that such models are developed across highly heterogeneous datasets and methods and are frequently not benchmarked head-to-head against conventional triage tools, with recurrent concerns about methodological quality and reporting. More broadly, the trade-off between sensitivity and specificity, and correspondingly between under-triage and over-triage, is a central consideration when AI/ML models are positioned against established triage scales, yet it was not systematically explored in our included studies.

Generalizability and External Validation

The generalizability of these models beyond their development settings remains largely unproven. All included studies used retrospective data from single or, in one case [17], two centers, with substantial variability in patient populations (adult, pediatric, mixed), healthcare systems (Iceland, Turkey, Taiwan, South Korea, USA), and data availability. Models developed on Taiwanese EHR data, for instance, may not generalize to US EDs with different documentation practices, triage workflows, or case mixes. A large-scale external validation by Shen et al. [30], which tested an ML prediction model across a broad inpatient population, illustrates how predictive performance and generalizability cannot be assumed when a model is applied beyond its development setting and must instead be demonstrated explicitly through external validation. None of our included studies undertook this kind of multi-site external transportability assessment.

In addition, the risk of bias assessment using PROBAST revealed that while six studies had low overall bias, one was unclear [17] and one was high risk [18], primarily due to deficiencies in the analysis domain. Common issues included a lack of calibration reporting, insufficient handling of missing data, and potential overfitting. These methodological shortcomings are consistent with broader evidence; Christodoulou et al. [31], in a systematic review of 71 studies and 282 model comparisons, found no consistent performance advantage of ML over LR and documented widespread methodological and reporting deficiencies, including failure to report model calibration in roughly four-fifths of studies. These findings emphasize the need for adherence to the Transparent Reporting of a Multivariable Prediction Model for Individual Prognosis or Diagnosis (TRIPOD) and PROBAST guidelines in future AI/ML triage research.

Implementation Gap and Future Directions

None of the included studies reported on prospective implementation or clinical impact. Model performance in retrospective datasets does not guarantee improved patient outcomes or safe real-world deployment. A randomized clinical trial by Wijnberge et al. [32], the Hypotension Prediction (HYPE) trial, showed that an ML-derived early warning system, when integrated into intraoperative care, reduced the depth and duration of hypotension, demonstrating that prospective evaluation of ML systems is both feasible and capable of changing clinically relevant outcomes; at the same time, the real-world benefit of any such system remains contingent on workflow integration and clinician adherence rather than predictive accuracy alone. These considerations underscore that successful implementation depends on workflow integration, clinician trust, alert fatigue management, and continuous monitoring rather than predictive accuracy alone.

Limitations

This systematic review has several limitations. First, the search was limited to four databases (PubMed, Scopus, Web of Science, and Embase), and only English-language publications were included, potentially introducing language and publication bias. Second, substantial clinical and methodological heterogeneity across the eight included studies, including differences in triage systems, outcome definitions, prediction horizons, and patient populations, precluded meta-analysis and necessitated a narrative synthesis. Third, all included studies were retrospective in design except one cross-sectional registry study, which inherently limits causal inference and may overestimate model performance due to temporal or selection biases. Fourth, the absence of prospective validation studies or clinical implementation trials in our review means that the real-world effectiveness and safety of these AI/ML models remain unknown. Fifth, potential publication bias favoring positive results cannot be excluded, as studies reporting poor or negative AI/ML performance may be less likely to be published. Sixth, despite using PROBAST for risk of bias assessment, inter-rater reliability was not formally evaluated, and the tool's emphasis on statistical reporting may penalize older or methodologically innovative studies that do not conform to contemporary reporting standards. 

## Conclusions

AI and ML models, particularly XGBoost and deep learning with NLP, can achieve high predictive accuracy for triage-related outcomes in ED settings, including multi-level triage urgency, hospital admission, ICU need, critical illness, and operational metrics such as length of stay. When directly compared, these models generally outperformed conventional triage scales such as the ESI, CTAS, and pedKTAS, suggesting that AI/ML may enhance risk stratification beyond current standard practices. However, the evidence base is limited by substantial methodological heterogeneity, lack of external validation, incomplete adherence to reporting guidelines, and the absence of prospective implementation studies. Most included studies were retrospective, and one-third had unclear or high risk of bias according to PROBAST, particularly due to deficiencies in calibration reporting and handling of missing data.

Future research should prioritize prospective, multicenter external validations, direct head-to-head comparisons with existing triage scales using decision curve analysis, adherence to TRIPOD and PROBAST standards, and, most critically, randomized controlled trials or rigorous implementation science studies to assess whether AI/ML-driven triage improves patient-important outcomes such as mortality, morbidity, and ED length of stay, without introducing unintended harms such as alert fatigue or algorithmic bias. Until such evidence emerges, AI/ML models for ED triage should be considered promising but experimental, warranting cautious integration into clinical workflows under continuous prospective monitoring.

## Appendices

**Table 4:** Detailed search strategy for each search engine

Database	Detailed search strategy
PubMed	(("Emergency Service, Hospital"[Mesh] OR "emergency department*"[tiab] OR "emergency room*"[tiab] OR ED[tiab] OR "emergency care"[tiab] OR triage[tiab]) AND ("Artificial Intelligence"[Mesh] OR "Machine Learning"[Mesh] OR "artificial intelligence"[tiab] OR AI[tiab] OR "machine learning"[tiab] OR "deep learning"[tiab] OR "neural network*"[tiab] OR "random forest"[tiab] OR "gradient boosting"[tiab] OR "logistic regression"[tiab] OR "predictive model*"[tiab]) AND (triage[tiab] OR prioritization[tiab] OR "risk stratification"[tiab] OR "patient acuity"[tiab]))
Scopus	TITLE-ABS-KEY (("emergency department*" OR "emergency room*" OR "emergency care" OR ED OR triage) AND ("artificial intelligence" OR AI OR "machine learning" OR "deep learning" OR "neural network*" OR "random forest" OR "gradient boosting" OR "logistic regression" OR "predictive model*") AND (triage OR prioritization OR "risk stratification" OR "patient acuity"))
Web of Science	TS=(("emergency department*" OR "emergency room*" OR "emergency care" OR ED OR triage) AND ("artificial intelligence" OR AI OR "machine learning" OR "deep learning" OR "neural network*" OR "random forest" OR "gradient boosting" OR "logistic regression" OR "predictive model*") AND (triage OR prioritization OR "risk stratification" OR "patient acuity"))
Embase	('emergency ward'/exp OR 'emergency department*':ti,ab OR 'emergency room*':ti,ab OR 'emergency care':ti,ab OR ED:ti,ab OR triage:ti,ab) AND ('artificial intelligence'/exp OR 'machine learning'/exp OR 'artificial intelligence':ti,ab OR AI:ti,ab OR 'machine learning':ti,ab OR 'deep learning':ti,ab OR 'neural network*':ti,ab OR 'random forest':ti,ab OR 'gradient boosting':ti,ab OR 'logistic regression':ti,ab OR 'predictive model*':ti,ab) AND (triage:ti,ab OR prioritization:ti,ab OR 'risk stratification':ti,ab OR 'patient acuity':ti,ab)
